# Plant-Based Alternative Products: Are They Healthy Alternatives? Micro- and Macronutrients and Nutritional Scoring

**DOI:** 10.3390/nu14030601

**Published:** 2022-01-29

**Authors:** Marcel Pointke, Elke Pawelzik

**Affiliations:** Division of Quality of Plant Products, Department of Crop Sciences, Faculty of Agriculture, University of Goettingen, 37075 Goettingen, Germany; epawelz@gwdg.de

**Keywords:** cheese alternative, meat alternative, micronutrients, Nutri-Score, online market analysis

## Abstract

In recent decades, the demand, supply, and consumption of plant-based (pb) alternative products have increased worldwide. The objective of this study was to characterize pb meat and cheese products and compare them with their respective animal-based products. Data were collected in online market analyses (2019/2021). Nutritional data, Nutri-Score, and analysis of micronutrients are presented in this article. The number of products has grown in all categories, with the largest increase of 110% in pb cheese. The main protein sources in pb meat were soy and wheat, followed by an increasing use of peas. Pb meat generally contained less energy and total and saturated fat, but more carbohydrates and sugars than meat. In pb cheese, the protein content was lower than that of cheese. In 3 of 17 food groups, the salt content of pb alternatives was lower than in animal products. The daily requirement for iron could be covered better by pb alternatives than previously anticipated as well as the need for the vitamins E and K. The calculated Nutri-Score was generally lower for pb meat and higher for pb cheese than for the respective animal products. The trend towards consumption of pb alternative products is increasing, but the high level of processing, wide range of nutrients, and high salt content indicate the need for nutritional guidelines for these products.

## 1. Introduction

From start-ups and leading companies to the world’s largest meat corporations, food manufacturers are developing fast-growing innovations in plant-based (pb) foods. This new generation of pb meat, fish, cheese, egg, and dairy products is increasingly competitive with animal products. Market research institutes conduct consumer studies to analyze, among other things, the motivations for consuming pb products. A German online survey [1] clarified that age is essential in individual motivation. More than 32% of the over-60 s stated that health reasons were their main motivation for abstaining from meat, while the 40–49 age group cited animal welfare reasons (27%). In the 18–29 age group, on the other hand, environmental and climate reasons (18%) play an important role. The discussion about reducing the consumption of animal-based foods has increased at many levels in recent years. The publication of the EAT–*Lancet* Commission, about a global planetary healthy diet [2], was decisive in the scientific field. In its latest report, the OECD/FAO [3] assumes that meat consumption in industrialized countries will not continue to rise as environmental and sustainability awareness increases in the population. Younger people, in particular, are increasingly adopting vegan, vegetarian, or flexitarian diets and thus reducing their consumption of animal products. In recent years, pb alternative products have experienced an enormous growth in the German and European food retail sector [4]. In Germany, for example, sales (€) of pb alternative products increased by 97% and sales volume (kg/L) increased by 80% between 2018 and 2020 [4].

Meat alternative products are produced based on proteins from plants (e.g., algae, cereals, legumes, mushrooms) or animal sources (e.g., eggs, insects, milk) [5,6,7,8]. Products made from insects currently still play a minor role in Western countries. Despite growing consumer interest in insects, there is still a large gap between curious sampling and actual acceptance. Numerous research groups are currently investigating the complexity of consumer acceptance of insects as food (food neophobia) and how this can be increased in the future [9,10,11]. Alternative cheese products are “cheese-like” products in which the main ingredient, milk, is replaced by plant components. Besides water, various vegetable edible fats/oils are usually the main ingredients. Other ingredients such as proteins and additives are blended into a homogeneous mixture using heat, mechanical shearing, and emulsifying salts [12,13]. The labeling of pb alternative products is currently being discussed on a social-political level in Europe. There are no specific regulations on these products, so the food information (FIC) regulation [14] applies. The basic principles of product information for consumers must be clear, concise, easy to understand, and not misleading. For better understanding, known products from food groups such as salami or mozzarella are referred to with the addition plant-based (pb) in this manuscript.

The extent to which alternative products can be compared with the respective animal products from a nutritional-physiological point of view is the main objective of the present study. Particular attention was paid to the micronutrients, as these are usually not declared by the manufacturers unless they are explicitly supplemented. With the help of the Nutri-Score, the mandatory information (“Big 7”) of the nutrition declaration [14] according to the manufacturer was used to obtain an overview of the macronutrients in the respective product group. With the agreement of numerous stakeholders, the Nutri-Score was introduced as a voluntary consumer information label (front-of-package nutrition labeling (FOPL)) in Germany in November 2020. Recent studies show that FOPL can have positive effects on shopping behavior by leading to, among other things, low consumption of products with a high energy, saturated fat, and sodium content [15,16,17,18]. Furthermore, consumers can more easily identify healthier products by replacing foods with red labeling with similar, currently available products without red labeling.

Satisfying the growing consumer demand for healthy, tasty, and sustainable foods, especially healthy alternatives to animal proteins, is a major challenge for the food industry. Therefore, this study aims to (I) provide an overview of the pb meat and cheese alternatives available in online stores in 2019 and 2021, and (II) characterize their nutrient composition, focusing on micronutrients compared to animal-based products, as well as the newly approved Nutri-Score as an FOPL.

## 2. Materials and Methods

### 2.1. Online Market Analysis

An online market analysis of pb meat and cheese alternative products was conducted in the first quarter of each of 2019 and 2021. As a result of the global COVID-19 pandemic, studies have shown that the eating habits of many consumers have changed, and meat consumption and overall consumption of animal-based products have decreased [19,20,21]. First-time buyers of pb meat were consumers who started to become flexitarians in the midst of the pandemic. Therefore, the second market analysis in 2021 will find out if and to what extent the variety of products has changed. Although products were offered directly by the manufacturer or the retailer on the websites, a list of ingredients and nutritional labeling (“Big 7”) had to be available there. Therefore, almost exclusively products available on the European market were included, as the legal regulations there are uniform, and the minimum information must be on the packaging [14]. In the internet search, keywords such as “meat alternative”, “cheese alternative”, “meat substitute”, “cheese substitute”, “meat-free”, “dairy-free”, “plant-based”, “vegan”, and “vegetarian” were used to cover as many available products as possible.

The study included products intended to mimic meat or cheese but made from primarily pb ingredients. The products were categorized according to their similarity to animal-based products. A distinction was also made between organic and conventional products, as there are guidelines for organic products in Europe [22] as to which ingredients may be added to a product. The two main differences in meat alternatives were the criterion of whether the food is eaten hot or cold. The first category (plant-based meat alternatives consumed hot, PBMA-hot) included product groups that were named “fillet”, “steak”, “schnitzel”, “burger”, “strips”, “minced”, “bratwurst”, or “sausage” in the product name or sales description. The second category included the following product groups (plant-based meat alternatives consumed cold, PBMA-cold): “meat sausage”, “salami”, “spreading sausage”, and “meat salad” (Table 1). The present study focused on “modern” meat alternative products, so products that did not specifically serve to imitate meat products, such as tofu, tempeh, and granulated products, were not considered. Products that were offered as alternatives to chicken meat or seafood were also not included. These are foods with a high degree of convenience according to the NOVA food classification [23], where consumers only need to perform a few preparation steps at home to consume them. Plant-based cheese alternatives (PBCA) were categorized into the product groups “sliced cheese”, “cheddar”, “cream cheese”, “mozzarella”, and “feta”. Pure nut products such as cashew and almond, which are fermented, were not included.

### 2.2. Data of Animal Foods from Databases

To make a comparison between plant- and animal-based products, data were collected from the following four national nutrient databases: Food Standards Australia New Zealand, AUSNUT [24]; Fineli, the Nutrition Unit of the National Institute for Health and Welfare in Finland [25]; the US Department of Agriculture (USDA) FoodData Central Data, Food and Nutrient Database for Dietary Studies 2017–2018 [26]; and the Max Rubner-Institute, Federal Research Institute of Nutrition and Food, Bundeslebensmittelschlüssel (BLS) Version 3.02 [27].

### 2.3. Nutri-Score

FOPL were created by a joint initiative of governments, product manufacturers, and retailers to encourage consumers to make healthier food choices by providing product information at a glance and attract attention [15]. The Nutri-Score is a color-coded, graded FOPL first introduced in France in 2017 [28] and has also been used in Germany on a voluntary basis since 2020. Classified foods can be divided into five categories by a nutritional score (from category A = dark green, indicating high nutritional quality, to category E = red, indicating low nutritional quality) [29]. In this study, the evaluation of nutrients and the calculation of the Nutri-Score were based on the calculation table of the Federal Ministry of Food and Agriculture (BMEL, German translation as of 2021). On a scale from −15 points (A) to +40 points (E), the nutrient content per 100 g of food was evaluated. Positive points (0–10) were assigned for dietary energy, total sugars, saturated fatty acids (SFA), and sodium. Negative points (0–5) were scored for fruits, vegetables, and nuts, fiber, protein, and canola, walnut, and olive oil content.

### 2.4. Sample Material

All products used for vitamin and mineral analysis in this study were purchased from online stores or local supermarkets. Four products with mostly different protein or fat sources were selected from each of the four product categories (meat sausage, salami, burger, sliced cheese) (Table 2).

### 2.5. Mineral and Vitamin Analysis

Freeze-dried material was used for mineral and vitamin analysis; for this purpose, the products had to be cut into pieces and freeze-dried (EPSILON 2-40; Christ, Osterode am Harz, Germany). Subsequently, the samples were ground with a coffee grinder (KSW 3307, Clatronic International, Kempen, Germany) and stored at +4 °C until analysis. Mineral concentrations were determined using a method adapted from that of Wheal et al. [30]. Approximately 100 mg of each sample was digested in 4 mL of 65% (*v/v*) nitric acid and 2 mL of 30% (*v/v*) hydrogen peroxide for 75 min at 200 °C and 40 bar in a microwave oven (Ethos 660; MWT AG, Heerbrugg, Switzerland). Samples were then made up to 25 mL with distilled water. Mineral concentrations were measured by inductively coupled plasma optical emission spectrometry (Vista-PRO CCD Simultaneous ICP-OES; Varian Inc., Palo Alto, CA, United States). Vitamin analysis of freeze-dried samples was conducted by bilacon (bilacon GmbH, Berlin, Germany, Department of Instrumental Analysis) using standardized procedures of the multi-method for determining water- and fat-soluble vitamins in food by LC-MS/MS (methods: PV-SA-158 and 159, 2019-02).

### 2.6. Statistical Analysis

SPSS^®^ statistical software (IBM SPSS Statistics, Version 26.0, Armond, NY, USA) and Microsoft Excel^®^ (Microsoft Office Professional Plus, 2013) were used for statistical analysis. One-way and two-way analysis of variance (ANOVA), followed by Tukey’s HSD test (*p* ≤ 0.05), were conducted to show significant differences.

## 3. Results

### 3.1. Market Analysis

In total, data were collected from 150 PBMA-hot products in 2019 and from 236 products in 2021 (+57%). Only a small proportion of this product category was labeled as organic. Compared to the earlier year, there were hardly any changes in organic products in this product category. The largest increase in samples from 2019 to 2021 was seen in the PBMA-cold category (from *n* = 48 to *n* = 101 or an increase of 110%). Furthermore, in this product category, significantly more than half of the products were produced conventionally. Overall, 65 cheese alternative products were included in the analysis in 2019 and 123 in 2021, representing an 89% increase in the number of products. The majority of the products available on the market were also not labeled as organic, but produced conventionally. However, the number of organic products increased significantly, by 140%, especially for alternative cream cheese products, by 260% (Table 3).

**Figure 1:**
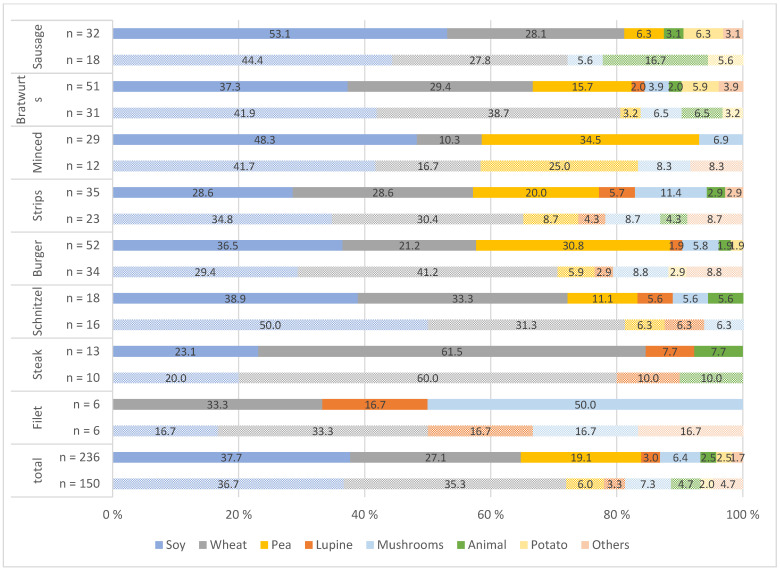
Protein sources used in plant-based meat alternatives (PBMA-hot) in 2019 (patterned color) and 2021 (solid color) (%); *n* = number of products; animal = milk or/and egg protein; others = beans, sunflower and pumpkin seeds.

**Figure 2:**
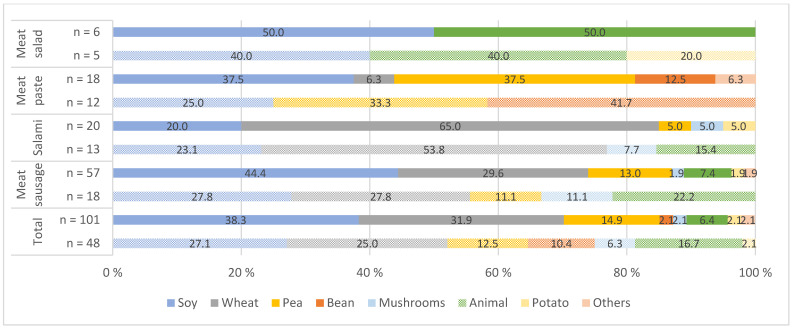
Protein sources used in plant-based meat alternatives (PBMA-cold) in 2019 (patterned color) and 2021 (solid color) (%); *n* = number of products; animal = milk and/or egg protein; others = millet, sunflower seeds.

**Figure 3:**
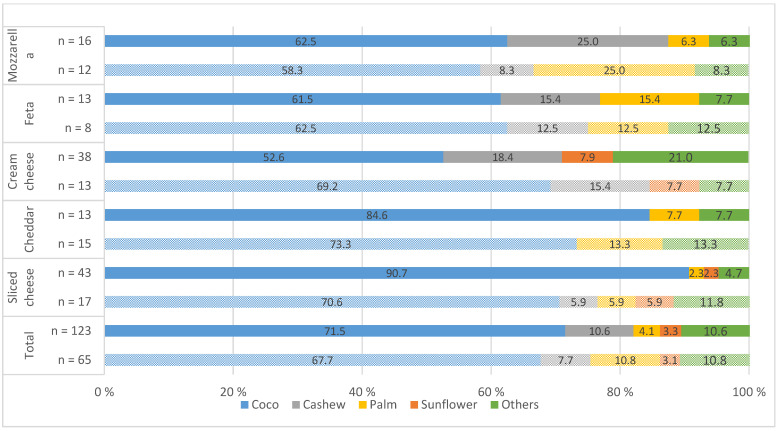
Fat sources used in plant-based cheese alternatives (PBCA) in 2019 (patterned color) and 2021 (solid color) (%); *n* = number of products; others = almond, shea butter, soy, olive, lupine.

The main protein sources of the investigated meat alternatives differed significantly depending on the product (Figure 1 and Figure 2). Soy was the most common protein source, and there was a year-to-year increase from 36.7% to 37.7% in PBMA-hot products and from 27.1% to 38.3% in PBMA-cold products. Wheat protein was the second most commonly used protein source. Overall, there was a clear increase in the use of pea protein, especially in PBMA-hot products, from 6.0% to 19.1%, and a significant decrease in animal proteins (milk protein and egg) in both pb product groups (PBMA-hot and PBMA-cold). Protein combinations were frequently found in the products. As shown in Figure 3, coconut oil was a common fat ingredient in the cheese alternatives. The use of palm oil was significantly reduced in 2021 from 10.8% to 4.1%. There was an increase in the use of cashew nuts, from 7.7% to 10.6%, and almonds, which accounted for 4.1% (Others) in 2021.

### 3.2. Nutrients and Nutri-Score

Table 4, Table 5 and Table 6 give an overview of the main nutrients in PBMA-hot, PBMA-cold, and PBCA products that were available as information for each of the categories studied. In addition, the Nutri-Score was calculated for all products. Since consumers can directly substitute animal meat and cheese products with pb alternative products, a comparison of nutrients and Nutri-Score was performed and included in the tables.

In the PBMA-hot product category (Table 4), the average energy value ranged from 152.5 to 244.4 kcal/100 g in 2019 and from 146.5 to 240.7 kcal/100 g in 2021. Fat content varied from 5.05 to 15.98 g/100 g, with sausage having the highest value in both years. The SFA content was 0.92 to 3.40 g/100 g, with the highest value for bratwurst (2019) and burger (2021) categories. Carbohydrate content was in the range from 4.34 to 15.48 g/100 g (2019) and from 4.91 to 16.88 g/100 g (2021), with the highest values in both years for schnitzel. The sugar content was 0.77–2.07 g/100 g in 2019 and 0.88–2.28 g/100 g in 2021, with the highest amounts in burger and steak, respectively. On average, a relatively high protein content, between 14.68 and 21.33 g/100 g, was observed in all product categories. Although the average salt content was generally less than 1.5 g/100 g, there was a wide range in the means of the product groups as well as from year to year, from 1.16 to 1.82 g/100 g, such that several products (2019 *n* = 7, 2021 *n* = 9) also contained more than 2.5 g of salt. In six out of a total of seven nutritional categories, fillet and strips had the lowest scores in both years. In the Nutri-Score calculation, fillet scored best, from −2.17 (2019) to −1.17 (2021), thus being in category A (dark green). Bratwurst and steak, on the other hand, achieved the highest score of 8.06 (2019) and 8.85 (2021), respectively, resulting in both of them being in category C (yellow).

Pb meat tends to have a lower kilocalorie content than animal-based meat (Table 4). In three out of eight food groups, namely burger, bratwurst, and sausage, the pb products showed significantly lower values than the animal-based products. A similar difference was found for total fat and SFA: their proportion was significantly higher in the animal-based products, except for minced meat (2021), where the SFA content in the pb products did not differ from that in animal-based products. Although, as expected, pb products contained more carbohydrates and sugars, there were no significant differences in PBMA-hot products compared to the animal variant except for burger. The salt content of pb products was significantly higher than for animal-based products in six out of eight food groups. The reverse was true for bratwurst, and there was no significant difference for meat sausage. The Nutri-Score was significantly better for the pb products in six categories. As with the pb sausage products, the animal-based sausage products received the highest scores, and the animal-based products scored lowest and were in category E (red).

The results for PBMA-cold products (Table 5) were similar, with the content of fat and SFA being significantly higher in animal-based products in two of the four product groups. The carbohydrate and sugar contents were significantly higher in all categories, twice as high for carbohydrates and four times as high for sugar, compared to animal products. The calculated Nutri-Score was overall lower in the pb products, from 7.50 to 14.92 in 2019 and 8.35 to 13.56 in 2021, corresponding to categories C and D (yellow/orange). The scores for meat products are significantly higher here in three out of four food groups.

Table 6 shows that for all product groups, the pb cheese alternatives had an energy content ranging from 249.0 to 288.2 kcal/100 g in a year-to-year comparison. In both years, the fat content was highest in the pb cream cheese products (24.72–24.78 g/100 g), and the SFA content was highest in the pb sliced cheese (16.27 to 18.14 g/100 g). Carbohydrate content ranged from 4.45 to 5.02 g/100 g in 2019 and from 20.19 to 21.09 g/100 g in 2021. The highest average protein values were found in pb feta in 2019 (6.84 g/100 g) and pb cream cheese in 2021 (5.72 g/100 g). The maximum salt content was calculated as 2.06 g/100 g for pb cheddar in 2019 and 2.02 g/100 g for sliced cheese in 2021. All cheese alternatives performed poorly in the calculated Nutri-Score, with cream cheese having the lowest score (11.62 to 14.13; category D (orange)); 20.13 points for pb cheddar (2019) and 21.07 points for sliced cheese (2021) would result in both being in category E (red). The Nutri-Score was significantly better than in animal products in four out of five product groups, while the protein content of the animal products was significantly higher in all product groups. As expected, the carbohydrate content was also significantly higher in pb products, except for cream cheese. The fat and SFA content did not differ significantly in the four food groups, regardless of whether the product was plant- or animal-based. Therefore, the low energy content in pb products can only be calculated significantly for sliced and cheddar cheese.

### 3.3. Minerals

Table 7 shows the mean percentage of the recommended daily intake according to the D-A-CH (Germany, Austria, Switzerland) reference values for the determined minerals, separated by gender and different age groups (19–25 years and ≥65 years). If no other recommendations are given for the male gender, the recommendations also apply to the female gender. The different age groups were chosen because the motivation to consume plant products may differ among age groups. The data for the animal products are based on the literature values. For the 19–25 age group (AG1+), zinc and iron in the alternative products were recalculated, a safety factor since it can be assumed that requirements for zinc of up to 50% higher and for iron of even 80% higher have to be met by a vegetarian diet rather than by a non-vegetarian diet [31]. Therefore, the daily requirement for iron, magnesium, copper, and sodium was better covered by pb products than by animal-based products. The same was true for calcium and phosphorus in the meat alternatives but not in the cheese alternatives. In the case of zinc content, the difference in product groups was noticeable. The calcium content by supplementation with calcium citrate was only increased in the product “Bedda”; with an addition of 700 mg/100 g this content is as high as in animal-based cheese products. This could be proven in the analysis. None of the other analyzed products contained any additional minerals.

### 3.4. Vitamins

The information on the water- and fat-soluble vitamins in Table 8 is to be read analogously to that for the minerals (Table 7). The requirement for vitamins B_1_, B_3_, and pantothenic acid is covered by all pb alternative products, to a lesser or equal extent than by animal-based products. In contrast, the need for vitamin B_6_ is met to a higher or equal extent. Pb cheese cannot meet the requirements for vitamin B_2_, folate, or biotin as well as pb meat alternatives. On the other hand, the cheese alternatives can cover up to 65.55% of the daily requirement for vitamin B_12_ and even up to 95.09% of that for vitamin C in females and 82.12% in males. According to the product declaration, only two alternative cheese products (Violife, Bedda) were supplemented with vitamin B_12_. The enrichment of vitamin B_12_ may explain the high value found. Vitamin C was added to the products in the form of ascorbic acid to extend shelf life. For the fat-soluble vitamins E and K, pb alternative products can cover the daily requirement equally well or significantly better than the animal-based alternatives, while vitamin A, however, was only present in small amounts. The detection of vitamin D was not possible in the group of pb products.

## 4. Discussion

Various studies have extensively proved that meat consumption has an impact on human and environmental health [2,32,33,34,35,36,37,38]. However, a conversion of Western nutrition habits, with a high portion of meat, on a global level would be possible over a longer period of time. Though the meat consumption per capita in Germany decreased overall by 750 g in 2020 compared to the previous year, especially for pork and beef, it still remains at a high level of 57.3 kg [39]. Different strategies for change are conceivable, such as smaller portions of meat from sustainable farming (“less, but better”) as well as greater consumption of vegetable proteins, where alternative products are relevant. Venti and Johnston [40] presented a vegetarian food pyramid with a subheading “Beans & Protein Foods”, in which they also mention meatless burgers and chicken and “nondairy” foods. In addition to soy milk and yogurt, soy cheese was also named [40]. The Giessen Vegan Food Pyramid also includes milk and yogurt alternatives, as well as “legumes and other protein sources”, which include tofu, lupine, and pea protein products [41]. Although pb foods such as tofu have been available for many years, they are currently not considered explicitly in country-specific guidelines [42] or documents such as that produced by the EAT–*Lancet* Commission [2].

### 4.1. Market Analysis

The data from the market analysis showed that the availability of pb alternative products has increased, and consumers are presented with a new variety of products when choosing their food. Especially with the rapid increase in new pb meat and cheese alternative products available, as shown in the market analysis (Table 3), the importance of these products in consumers’ daily food choices has increased. Recent studies have shown that shifting to a diet with a reduction or elimination of animal-based products to more whole-grain foods and pb foods has been one of the most important dietary strategies on a global scale for both the planet and human health [32,33,34,35,43]. A study by Kemper and White [44] showed that the pb product category’s dietary implications and individual diversity have gained importance as more people adopt flexitarian, vegetarian, and vegan dietary styles. Sundar and Kardes [45] described the “health halo effect”, through which consumers automatically perceive the variety of pb alternative products as healthier. The results of the present study, though, cannot confirm such theory, as the products studied here and most of the foods found in the market analysis have to be classified as ultra-processed foods according to the NOVA classification (Group 4) of Monteiro et al. [23]. These food products contain various ingredients, particularly additives such as dyes and other colors, flavors, flavor enhancers, and emulsifiers, and are produced through a number of different industrial processes. They are usually ready to heat and eat and are enticingly packaged and intensively marketed [23]. Gehring et al. [46] found in their study that a significant avoidance of animal foods was associated with greater consumption of ultra-processed foods. Thus, vegetarian/vegan diets are not necessarily beneficial to health, as studies have shown that consumption of ultra-processed foods can potentially negatively affect the nutrient quality and, thus, health outcomes [23,47]. The most important criteria for consumers in their purchasing and consumption decisions for meat alternatives were their sensory characteristics and the availability of the products, and only secondarily do animal welfare and environmental as well as health aspects influence consumers [48]. The number of alternative products has increased significantly from 2019 to 2021: by 57% for PBMA-hot, by 110% for PBMA-cold, and by 89% for PBCA. During this time, the products have evolved from niche products to mainstream products in German supermarkets, which often place them close to animal products. Dutch [49] and Australian [50] consumer studies investigated motivations for eating pb meat substitutes. They found that for switching to a pb sustainable diet, alternative products can be a valuable aid. Considering cultural and social factors, especially at family gatherings or other occasions where animal-based products are consumed, pb foods can provide a good alternative for consumers who prefer them [49,50]. Interestingly, consumer studies have shown that meat alternatives primarily appeal to consumers who want to replace meat in their meal, rather than consumers who identify themselves as vegetarians and vegans and are more likely to question the purpose of eating meat-like foods [50,51].

### 4.2. The “Big 7” of the Plant-Based Alternative Products

Considering the categorization of foods by their energy content (low: ≤150 kcal/100 g; medium: 160–240 kcal/100 g; high: ≥250 kcal/100 g [52]), only the product group “fillet” had a low energy content in 2021. In the PBMA-hot category (Table 4), all other product groups had a medium energy content (160–240 kcal/100 g). A high energy content (≥250 kcal/100 g) was observed in two of the four PBMA-cold product groups (Table 5). For the PBCA category (Table 6), all products had a high energy content. However, compared to the animal-based products, in the three categories (PBMA-hot, PBMA-cold, PBCA), the energy content of the alternative products was significantly lower in burger, bratwurst, sausage, salami, sliced cheese, and cheddar. Since the global obesity epidemic is linked to excessive daily energy intake [53], low energy density foods should also be consumed to prevent secondary diseases such as cardiovascular diseases (CVDs) or cancer [36,54].

The total fat and SFA content in the meat alternatives was not substantial or significantly lower than in the animal products. It was noticeable that the amount of fat can vary considerably within all product categories. A high proportion of ultra-processed foods with a high energy density can also be associated with excessive fat intake in the long term [23]. In both years, the market analysis for pb cheese in Figure 3 shows that the main fat source for all products was coconut oil: 76.7% in 2019 and 71.5% in 2021. The high content of SFA in coconut oil may have implications for elevated blood concentrations of total and LDL cholesterol. Associations between coconut oil consumption and the risk of CVDs are controversially discussed in studies [55,56].

The present study shows that the main protein sources in meat alternatives are soy, wheat, and pea (Figure 1 and Figure 2). The quality of dietary proteins can be determined by the protein digestibility corrected amino acid score (PDCAAS). The PDCAAS for milk and whey protein concentrate, soy protein isolate, and egg were rated at the highest possible score of 1.0, whereas pea protein concentrate was rated at 0.89. Wheat and wheat gluten had the lowest scores with 0.51 and 0.25, respectively. In comparison, red meat was rated at 0.92 [57,58]. Proteins of plant origin are often deficient in one or more essential amino acids. They are less digestible in their natural form than animal proteins due to antinutritional compounds, such as phytic acid [59]. Nevertheless, dietary intake of plant protein may be more positively evaluated than that of animal protein due to its potential health benefits. The studies by Shang et al. [37] and Song et al. [38] showed that a higher intake of plant protein tends to be associated with a low risk of type 2 diabetes and of all-cause and cardiovascular mortality. Accordingly, the protein quality of pea-, soy-, milk-, and/or egg-based meat alternatives was comparable to that of beef in terms of essential amino acids, but wheat- or gluten-based products had a lower protein quality than comparable meat products. In addition, many products contain combinations of multiple protein sources, and thus protein quality can be improved. The protein content of each meat alternative product, as well as of each category, sometimes differs significantly. For example, two PBMA-hot categories (bratwurst, sausage) had a considerably higher protein content than the respective animal category. On the other hand, the protein content in all five cheese groups was substantially lower than in the animal-based products (see Table 6), so that pb cheese products currently do not offer an adequate protein alternative.

The results of the market analysis showed that the salt content in pb meat alternatives was significantly higher than in the respective meat product. Recommendations for salt reduction explicitly for meat alternatives were presented by Public Health England in 2020 [60]. Three subgroups were classified from that study [60] and recommendations for the salt content were made based on 100 g of product as follows: “plain meat alternatives” (e.g., fillets, mince) with a low salt content of 0.63 g/100 g, “meat-free products” (sausage, burger) with a medium salt content of 1.19 g/100 g, and “meat-free bacon” (cold cuts) with a high salt content of 1.78 g/100 g. Comparison of the product categories showed that all PBMA-hot products were above the maximum recommended value. In the PBMA-cold category, there were three products in 2019 and one in 2021 that did not exceed the value of 1.78 g/100 g (Table 5). The mineral analysis (Table 7) found significantly higher sodium levels in all the pb products analyzed here, compared to the data from the food databases [24,25,26,27] for the animal-based products. Especially in the products pb salami and pb sausage, the recommended daily intake was covered or even exceeded with 100 g of food. However, it should be noted that the consumer adds salt or sodium-containing seasonings to the less processed animal-based product (e.g., fillet, steak, minced meat) but not to the pb convenience product. Because of such additions, similar high sodium levels could be achieved as in the vegetable alternatives in this study. These results indicate the need for industry guidelines.

### 4.3. Nutri-Score

On calculating the Nutri-Score for alternative and animal-based products, significant differences were found. In PBMA-hot (Table 4) and PBMA-cold (Table 5), significantly higher scores were calculated for the meat products in nine of the twelve product groups, mainly due to the high proportion of SFA and salt. The results for cheese alternatives were the opposite; in four out of five product groups (Table 6), the pb alternatives had a higher Nutri-Score than the animal products. This can be explained by the very low protein content of pb products. That the Nutri-Score can be an effective tool to inform consumers and make healthier purchasing decisions has been shown in recent studies [15,16]. Therefore, manufacturers should increasingly declare this FOPL on their products. The Nutri-Score is colored like a traffic light and makes it easy for consumers to understand which product is the “healthier” one. However, it is not able to reflect the degree to which the products are processed. As shown by Romero Ferreiro et al. [61], there were ultra-processed foods in each Nutri-Score category (A–E), oriented according to the NOVA classification. According to their results, in category B, more than half (51.5%) of the products were highly processed foods. The prospective French cohort study, NutriNet-Santé, was able to show that ultra-processed foods have negative effects on various diseases, such as associations with a higher risk of CVD [62] and depressive symptoms [63], and Schnabel et al. [47,64] observed effects with gastrointestinal disease and an overall higher risk of mortality. Given the negative impact that consumption of ultra-processed foods has on various aspects of health, FOPL with the Nutri-Score should be followed with at least additional labeling indicating the degree of processing, such as the NOVA classification. In this way, it becomes clear that each pb alternative product has individually different product characteristics and these different aspects have to be evaluated in order to classify a food as “healthy”.

### 4.4. Micronutrients of the Plant-Based Alternative Products

The enrichment of pb foods with micronutrients is also becoming increasingly important. The more products are offered and the greater their acceptance among consumers, the more important it becomes to have a uniform European regulation to achieve nutritional equivalence compared to animal-based products in order to avoid possible deficits in calcium, iron, zinc, and vitamin B_12_ in certain population groups [65]. The mineral and vitamin analyses in Table 7 and Table 8 show that most of the pb cheese and meat alternatives meet the daily nutritional recommendations for single micronutrients.

Iron in plant foods has generally a lower bioavailability than iron in meat, because some of the iron in meat is bound to hemoglobin (heme iron), which is a more bioavailable form of iron than non-heme iron [31,66]. In the present study, the iron content of the plant alternatives was higher than the reference data from the literature [24,25,26,27] for animal products. Even after calculating a safety factor of +80%, the results are the same or higher. The same results can be seen in Table 7 for the male gender and the age groups (AG1, AG1+). As the D-A-CH reference values for iron in older women (≥65 years) are lowered, the daily recommendations can be reached faster (AG2). Thus, the products selected here may provide a good alternative.

In contrast, except for the salami alternatives, the zinc content of the products was significantly lower than the average literature references for animal-based products from the databases [24,25,26,27] and, thus, did not represent an adequate zinc alternative, especially when the safety factor was taken into account. In addition, antinutritional substances such as phytic acid may reduce zinc absorption in pb alternatives [67,68].

In an omnivorous diet, milk and dairy products, especially hard cheese, are an essential source of calcium [68]. The results of the present study showed that on average 100 g of sliced cheese covers 76% of the requirement, whereas the vegetable alternatives provide only 32% (Table 7). Such a high content can only be achieved if the manufacturer fortifies the product with calcium. On the other hand, meat alternatives also appear to be a source of calcium, as they can cover the requirement much better than meat-based products. Other important sources of calcium are green leafy vegetables and calcium-rich mineral water [68].

Vitamin B_12_ was found exclusively in animal products. Vegetarians who consume milk, cheese, and eggs can get a sufficient supply. Vegans rely on enriched foods or supplements such as toothpaste containing vitamin B_12_ to meet their needs [69,70]. However, only conventional foods can be supplemented with vitamins because it is not legally permitted for organic products in Europe [22]. Therefore, it would be beneficial if pb alternative products contained this nutrient. In the present study, only the cheese alternatives were able to meet the recommended daily intake to the same extent as the animal-based products, as the meat alternatives contain only very low levels of B_12_. The reason for this was the producer adding vitamin B_12_ to the PBCA.

To our knowledge, the present study was the first to focus solely on pb products available in online stores and to compare them with animal-based products and calculate the Nutri-Score for these products. However, due to the large number and variety of “modern” pb alternative products on the market, the results obtained in this study cannot be generalized in principle. Nevertheless, the selection of products from online stores represents a broad part of the market for meat and cheese alternatives, so that information and recommendations can be derived from it.

## 5. Conclusions

The trend towards pb alternative products is rising, especially in the Western world. These alternative products mainly provide high-quality vegetable protein. The present study showed that the content of fat, SFA, and salt in the pb products varied considerably. These nutrients are related to the most important dietary factor in the global burden of disease. The results of the micronutrient assessment show that the relationship to the reference values for the recommended daily intake is meaningful. In this way, deficiencies and surpluses of vitamins and minerals can be made clear. Due to the high degree of processing of the foods studied here, they can nevertheless not be recommended for the daily diet, even if they have a low Nutri-Score. However, consumers should also pay attention to the nutrition labeling of the individual alternative products, as these do not automatically represent a healthier alternative to an animal-based product. Therefore, intervention studies would be of particular interest to clarify whether substituting animal-based foods with pb alternatives has an impact on health. Furthermore, consumers need guidance on how to compose a balanced pb diet.

## Figures and Tables

**Table 1:** Classification of pb alternative product categories.

Category		Description
PBMA-hot	Fillet	Either contains “tenderloin” in the product name or is a meat-free product that appears to imitate beef/pork tenderloin
	Steak	Either contains “steak” in the product name or is a meat-free product that appears to imitate beef/pork steak
	Schnitzel	Either contains “schnitzel” in the product name or is a meat-free product that appears to imitate breaded meat
	Burger	Either contains “burger” and/or “pattie/patty” in the product name or is a meat-free product that appears to imitate beef burger
	Strips	Either contains “gyros”, “chunks”, and/or “strips” in the product name or is a meat-free product that appears as small thin slices or strips
	Minced meat	Either contains “mince” in the product name or is a meat-free product that appears to imitate minced meat
	Bratwurst	Either contains “bratwurst” or “barbecue sausage” in the product name or is a meat-free product that appears to imitate bratwurst
	Sausage	Either contains “Wiener”, “Frankfurter”, and/or “Hot Dog” in the product name or is a meat-free product that appears to imitate sausage
PBMA-cold	Meat sausage	Either contains “Lyoner”, “Mortadella”, and/or “cold cuts” in the product name or is a meat-free product that appears to imitate meat sausage, which can be used in sandwiches
	Salami	Either contains “salami” in the product name or is a meat-free product that appears to imitate salami, which can be used in sandwiches
	Spreading sausage	Either contains “liver sausage” and/or “pâté” in the product name or is a meat-free product that appears to imitate spreading sausage, which can be used in sandwiches
	Meat salad	Meat-free product that appears to imitate meat salad, which can be used in sandwiches
PBCA	Sliced cheese	Either contains “Gouda” in the product name or is a dairy-free product that appears to imitate sliced cheese, which can be used in sandwiches
	Cheddar	Either contains “Cheddar” in the product name or is a dairy-free product that appears to imitate cheddar, which can be used in sandwiches
	Cream cheese	Either contains “fresh” and/or “cream” in the product name or is a dairy-free product that appears to imitate cream cheese, which can be used in sandwiches
	Mozzarella	Either contains “mozzarella” in the product name or is a dairy-free product that appears to imitate mozzarella, which can be used in sandwiches
	Feta	Either contains “Greek style” in the product name or is a dairy-free product that appears to imitate brined cheese or feta, which can be used in sandwiches

PBMA-hot = plant-based meat alternatives consumed hot; PBMA-cold = plant-based meat alternatives consumed cold; PBCA = plant-based cheese alternatives; product name also refers to the sales description.

**Table 2:** Pb products for vitamin and mineral analysis.

Category		Product Name (German Name)		Manufacturer or Distributor
PBMA-cold	Meat sausage	Vegan cold cuts	(Veganer Aufschnitt Lyoner Art)	Heirler
		Vegan cold cuts	(Vegan Aufschnitt Natur)	Veganz
		Vegan cold cuts	(Veganer Aufschnitt auf Basis von Pflanzenprotein nach Lyoner Art)	EDEKA
		Vegetarian cold cuts	(Vegetarischer Schinken Spicker Mortadella)	Rügenwalder
	Salami	Vegan salami	(Veganer Aufschnitt Salami Art)	Heirler
		Vegan salami	(Aufschnitt Rustikal nach Salami Art)	Hobelz
		Vegan salami	(Veganer Aufschnitt nach Art Salami)	EDEKA
		Vegetarian salami	(Vegetarische Mühlen Salami Klassisch)	Rügenwalder
PBMA-hot	Burger	Vegetarian burger patty	(Classic Burgers) (frozen)	Quorn
		Vegan burger patty	(Incredible Burger) (chilled)	Garden Gourmet
		Vegan burger patty	(Next Level Burger) (chilled)	Lidl
		Vegan burger patty	(Beyond Burger) (frozen)	Beyond Meat
PBCA	Sliced cheese	Vegan cheese	(Original Geschmack Scheiben)	Violife
		Vegan cheese	(Scheiben Classic)	Bedda
		Vegan cheese	(Natur Genießerscheiben)	SimplyV
		Vegan cheese	(Mr. Berta Schmelz Scheiben)	Soyatoo!

PBMA-hot = plant-based meat alternatives consumed hot; PBMA-cold = plant-based meat alternatives consumed cold; PBCA = plant-based cheese alternatives.

**Table 3:** Development of the number and type of products of pb cheese and meat alternatives, and the distribution (%) of products with organic labels between 2019 and 2021.

Category		Pb Products (*n*)				Increase from 2019 to 2021 (%)	
		2019		2021			
		Total	Organic	Total	Organic	Total	Organic
PBMA-hot	Fillet	6	4	6	4	0	0
	Steak	10	8	13	8	30	0
	Schnitzel	16	6	18	2	13	−67
	Burger	34	17	52	14	53	−18
	Strips	23	13	35	16	52	23
	Minced	12	4	29	4	142	0
	Bratwurst	31	13	51	19	65	46
	Sausage	18	7	32	13	78	86
	**Total**	**150**	**72**	**236**	**80**	**57**	**11**
PBMA-cold	Meat sausage	18	5	57	18	217	260
	Salami	13	6	20	8	54	33
	Spreading sausage	12	6	18	7	50	17
	Meat salad	5	1	6	2	20	100
	**Total**	**48**	**18**	**101**	**35**	**110**	**94**
PBCA	Sliced cheese	17	4	43	4	153	0
	Cheddar	15	1	13	1	−13	0
	Cream cheese	13	5	38	18	192	260
	Mozzarella	8	3	13	6	63	100
	Feta	12	3	16	7	33	133
	**Total**	**65**	**15**	**123**	**36**	**89**	**140**

PBMA-hot = plant-based meat alternatives—consumed hot; PBMA-cold = plant-based meat alternatives—consumed cold; PBCA = plant-based cheese alternatives.

**Table 4:** Nutrients (“Big 7”) and Nutri-Score per 100 g of pb meat alternatives and meat (mean and standard deviation) in eight different food groups, compared to animal-based products *.

**Nutrient Criteria**	**Pb Fillet** **2019 (*n* = 6)**				**Pb Fillet** **2021 (*n* = 6)**				**Meat Fillet** **(*n* = 15)**				**Pb Steak** **2019 (*n* = 10)**				**Pb Steak** **2021 (*n* = 13)**				**Meat Steak** **(*n* = 73)**				**Pb Schnitzel** **2019 (*n* = 16)**				**Pb Schnitzel** **2021 (*n* = 18)**				**Meat Schnitzel** **(*n* = 19)**			
Energy (kcal)	152.5	±	75.6		146.5	±	63.5		141.6	±	32.6		188.0	±	54.9		206.6	±	54.2		182.4	±	131.3		244.4	±	38.8		240.7	±	41.4		224.3	±	42.9	
Fat (g)	5.58	±	3.89		5.05	±	3.81		5.74	±	3.47		7.52	±	4.79		9.13	±	4.30		10.69	±	16.36		11.98	±	3.53		11.76	±	3.95		10.88	±	4.85	
Saturated fat (g)	0.92	±	0.78		0.92	±	0.79		2.14	±	1.21		1.76	±	2.28		2.75	±	2.81		4.09	±	6.34		1.78	±	1.67	^a^	1.34	±	0.58	^a^	3.62	±	1.62	^b^
Carbohydrate (g)	5.67	±	3.50	^b^	5.22	±	2.55	^b^	0.00	±	0.00	^a^	8.14	±	5.98	^b^	8.17	±	5.53	^b^	0.01	±	0.05	^a^	15.48	±	4.48	^b^	16.88	±	4.78	^b^	9.13	±	5.08	^a^
Sugars (g)	0.77	±	0.75	^b^	0.88	±	0.57	^b^	0.00	±	0.00	^a^	1.38	±	0.86	^b^	2.28	±	1.81	^c^	0.00	±	0.00	^a^	1.65	±	0.79	^b^	1.63	±	1.29	^b^	0.44	±	0.32	^a^
Protein (g)	17.52	±	9.96		18.00	±	8.15		22.36	±	2.02		20.46	±	6.90		20.95	±	6.58		21.61	±	4.23		16.69	±	4.58	^a^	14.68	±	3.75	^a^	22.22	±	2.41	^b^
Salt (g)	1.19	±	0.45	^b^	1.22	±	0.52	^b^	0.22	±	0.24	^a^	1.57	±	0.51	^b^	1.82	±	0.35	^b^	0.21	±	0.31	^a^	1.71	±	0.43	^b^	1.55	±	0.30	^b^	0.46	±	0.27	^a^
Nutri-Score	−2.17	±	7.49		−1.17	±	9.02		4.33	±	7.18		3.90	±	6.84		8.85	±	7.07		5.37	±	7.38		4.88	±	7.02	^a^	3.50	±	5.31	^a^	10.47	±	7.00	^b^
**Nutrient Criteria**	**Pb Burger** **2019 (*n* = 34)**				**Pb Burger** **2021 (*n* = 52)**				**Meat Burger** **(*n* = 26)**				**Pb Strips** **2019 (*n* = 23)**				**Pb Strips** **2021 (*n* = 35)**				**Meat Strips** **(*n* = 21)**				**Pb Minced** **2019 (*n* = 12)**				**Pb Minced** **2021 (*n* = 29)**				**Meat Minced** **(*n* = 21)**			
Energy (kcal)	223.0	±	55.1	^ab^	203.5	±	42.5	^a^	236.2	±	32.7	^b^	163.3	±	68.1		195.2	±	52.3		205.8	±	121.1		173.4	±	56.9		177.6	±	41.2		199.4	±	45.6	
Fat (g)	9.90	±	5.13	^a^	11.32	±	4.25	^a^	14.32	±	3.75	^b^	6.87	±	5.45	^a^	9.33	±	4.38	^ab^	13.40	±	13.42	^b^	6.44	±	4.84	^a^	8.59	±	4.36	^a^	12.00	±	4.47	^b^
Saturated fat (g)	2.09	±	2.38	^a^	3.40	±	3.65	^a^	5.51	±	1.28	^b^	1.27	±	1.09	^a^	1.78	±	1.63	^a^	4.79	±	5.76	^b^	0.98	±	1.00	^a^	3.26	±	3.70	^b^	4.92	±	1.75	^b^
Carbohydrate (g)	11.42	±	7.23		8.94	±	5.83		7.72	±	8.22		4.34	±	2.84	^ab^	4.91	±	2.28	^b^	2.56	±	3.96	^a^	6.19	±	4.28	^b^	7.76	±	6.84	^b^	1.43	±	2.61	^a^
Sugars (g)	2.07	±	1.54		1.60	±	1.54		1.32	±	1.50		1.10	±	1.05		1.40	±	1.22		0.91	±	1.51		1.34	±	0.96	^b^	1.24	±	1.28	^b^	0.23	±	0.42	^a^
Protein (g)	17.23	±	8.06	^ab^	15.28	±	5.34	^a^	18.82	±	4.03	^b^	19.77	±	7.99		21.33	±	6.01		18.43	±	7.21		21.18	±	7.88	^b^	16.73	±	4.37	^a^	21.24	±	2.91	^b^
Salt (g)	1.48	±	0.61	^b^	1.44	±	0.41	^b^	1.01	±	0.49	^a^	1.16	±	0.61	^ab^	1.47	±	0.75	^b^	0.85	±	1.20	^a^	1.25	±	0.63	^b^	1.26	±	0.55	^b^	0.40	±	0.39	^a^
Nutri-Score	4.50	±	5.70	^a^	5.27	±	6.75	^a^	13.54	±	6.37	^b^	0.61	±	6.95	^a^	3.23	±	7.82	^a^	8.86	±	8.96	^b^	−0.50	±	6.30	^a^	3.03	±	7.10	^a^	8.10	±	5.76	^b^
**Nutrient Criteria**	**Pb Bratwurst** **2019 (*n* = 31)**				**Pb Bratwurst** **2021 (*n* = 51)**				**Meat Bratwurst** **(*n* = 28)**				**Pb Sausage** **2019 (*n* = 18)**				**Pb Sausage** **2021 (*n* = 32)**				**Meat Sausage** **(*n* = 26)**															
Energy (kcal)	224.7	±	44.1	^a^	218.6	±	45.0	^a^	267.6	±	41.0	^b^	229.1	±	45.4	^a^	224.8	±	34.8	^a^	264.0	±	67.2	^b^												
Fat (g)	13.43	±	5.58	^a^	13.62	±	4.80	^a^	22.14	±	5.00	^b^	15.98	±	4.93	^a^	13.97	±	3.44	^a^	21.95	±	7.94	^b^												
Saturated fat (g)	2.66	±	2.23	^a^	3.05	±	2.87	^a^	8.84	±	1.85	^b^	1.57	±	0.77	^a^	2.39	±	2.85	^a^	8.47	±	2.97	^b^												
Carbohydrate (g)	5.41	±	2.94	^b^	6.00	±	2.87	^b^	2.16	±	2.40	^a^	5.10	±	2.53	^b^	5.32	±	2.28	^b^	2.47	±	2.55	^a^												
Sugars (g)	1.46	±	1.42	^b^	1.42	±	1.22	^b^	0.53	±	0.36	^a^	1.04	±	0.68	^b^	1.40	±	0.90	^b^	0.52	±	0.62	^a^												
Protein (g)	19.80	±	6.26	^b^	17.12	±	6.68	^ab^	15.08	±	1.56	^a^	15.07	±	6.96	^ab^	18.91	±	7.50	^b^	13.24	±	2.12	^a^												
Salt (g)	1.76	±	0.40	^a^	1.66	±	0.36	^a^	1.98	±	0.37	^b^	1.75	±	0.66		1.77	±	0.85		1.76	±	0.40													
Nutri-Score	8.06	±	5.13	^a^	7.45	±	5.68	^a^	23.21	±	4.08	^b^	7.67	±	5.19	^a^	7.94	±	4.72	^a^	20.04	±	3.77	^b^												

* Calculated mean values from AUSNUT, Fineli, USDA, and BLS databases; ^a–c^ values within a food group with differing superscript letters are statistically significantly different (*p* ≤ 0.05).

**Table 5:** Nutrients (“Big 7”) and Nutri-Score per 100 g of pb meat cold cuts alternatives and cold cuts meat (mean and standard deviation) in four different food groups, compared to animal-based products *.

**Nutrient Criteria**	**Pb Meat Sausage** **2019 (*n* = 18)**				**Pb Meat Sausage** **2021 (*n* = 57)**				**Meat Sausage** **(*n* = 37)**				**Pb Salami** **2019 (*n* = 13)**				**Pb Salami** **2021 (*n* = 20)**				**Meat Salami** **(*n* = 23)**				**Pb Spreading Sausage** **2019 (*n* = 12)**				**Pb Spreading Sausage** **2021 (*n* = 18)**				**Meat Spreading Sausage** **(*n* = 16)**			
Energy (kcal)	185.4	±	49.1		198.1	±	45.6		215.6	±	84.4		210.3	±	37.7	^a^	218.3	±	46.9	^a^	364.4	±	75.0	^b^	280.7	±	53.5		276.3	±	43.0		293.1	±	72.5	
Fat (g)	10.82	±	5.20	^a^	12.05	±	4.29	^a^	16.04	±	9.48	^b^	10.06	±	3.03	^a^	11.83	±	4.92	^a^	30.54	±	8.65	^b^	24.68	±	7.18		23.32	±	5.27		24.40	±	9.87	
Saturated fat (g)	2.45	±	2.91	^a^	1.92	±	1.57	^a^	6.00	±	3.57	^b^	1.67	±	1.31	^a^	2.21	±	2.08	^a^	11.23	±	3.11	^b^	11.86	±	9.10		9.08	±	8.24		9.53	±	4.01	
Carbohydrate (g)	4.95	±	3.48	^b^	5.21	±	3.32	^b^	2.96	±	2.89	^a^	6.53	±	2.83	^b^	5.86	±	2.12	^b^	0.97	±	0.94	^a^	6.69	±	3.94		8.11	±	4.44		3.87	±	6.57	
Sugars (g)	1.85	±	1.26	^b^	1.73	±	0.94	^b^	1.73	±	0.94	^a^	1.88	±	0.99	^b^	2.07	±	1.20	^b^	0.52	±	0.42	^a^	1.91	±	1.55	^b^	1.81	±	0.85	^b^	0.68	±	0.54	^a^
Protein (g)	16.19	±	9.44		16.34	±	8.94		14.51	±	5.43		21.94	±	9.23		20.44	±	10.57		21.50	±	2.56		6.82	±	2.87	^a^	6.61	±	3.64	^a^	14.69	±	3.87	^b^
Salt (g)	1.76	±	0.81		1.96	±	0.75		2.23	±	0.98		2.17	±	0.74	^a^	2.25	±	0.60	^a^	3.47	±	0.70	^b^	1.70	±	0.34		1.80	±	0.31		1.71	±	0.78	
Nutri-Score	7.50	±	8.78	^a^	8.35	±	6.12	^a^	19.51	±	5.02	^b^	8.85	±	5.46	^a^	10.45	±	3.62	^a^	25.91	±	2.98	^b^	14.92	±	7.2	^a^	13.56	±	7.34	^a^	20.50	±	2.85	^b^
**Nutrient Criteria**	**Pb Meat Salad** **2019 (*n* = 5)**				**Pb Meat Salad** **2021 (*n* = 6)**				**Meat Salad** **(*n* = 11)**																											
Energy (kcal)	262.0	±	27.1		274.2	±	28.8		237.5	±	77.5																									
Fat (g)	24.08	±	3.71		24.92	±	4.11		20.38	±	9.44																									
Saturated fat (g)	2.28	±	0.65		5.10	±	6.34		6.30	±	3.09																									
Carbohydrate (g)	5.78	±	1.36		5.52	±	0.79		3.14	±	3.22																									
Sugars (g)	4.66	±	1.10	^b^	4.70	±	0.92	^b^	0.76	±	0.64	^a^																								
Protein (g)	4.56	±	3.33	^a^	6.22	±	3.57	^ab^	10.53	±	4.22	^b^																								
Salt (g)	1.53	±	0.46		1.26	±	0.41		1.74	±	0.80																									
Nutri-Score	10.00	±	3.67		10.7	±	6.02		15.55	±	5.89																									

* Calculated mean values from AUSNUT, Fineli, USDA, and BLS databases; ^a,b^ values within a food group with differing superscript letters are statistically significantly different (*p* ≤ 0.05).

**Table 6:** Nutrients (“Big 7”) and Nutri-Score per 100 g of pb cheese alternatives and cheese (mean and standard deviation) in five different food groups, compared to animal-based products *.

**Nutrient Criteria**	**Pb Sliced Cheese** **2019 (*n* = 17)**				**Pb Sliced Cheese** **2021 (*n* = 43)**				**Sliced Cheese** **(*n* = 53)**				**Pb Cheddar** **2019 (*n* = 15)**				**Pb Cheddar** **2021 (*n* = 13)**				**Cheddar** **(*n* = 25)**				**Pb Cream Cheese** **2019 (*n* = 13)**				**Pb Cream Cheese** **2021 (*n* = 38)**				**Cream Cheese** **(*n* = 32)**			
Energy (kcal)	286.2	±	47.2	^a^	283.3	±	13.7	^a^	339.4	±	56.8	^b^	279.5	±	52.5	^a^	288.2	±	21.5	^ab^	329.2	±	72.9	^b^	268.5	±	46.7		267.7	±	38.4		256.5	±	73.6	
Fat (g)	22.14	±	4.60	^a^	21.62	±	1.76	^a^	26.35	±	6.51	^b^	20.93	±	4.50		21.92	±	2.65		24.49	±	9.13		24.72	±	5.62		24.78	±	3.63		22.79	±	9.24	
Saturated fat (g)	16.27	±	4.67		18.14	±	3.04		16.77	±	4.24		15.32	±	5.69		18.05	±	3.63		15.73	±	5.71		15.94	±	8.73		14.97	±	8.20		14.45	±	5.96	
Carbohydrate (g)	19.58	±	4.88	^ab^	21.09	±	2.06	^b^	0.78	±	1.22	^a^	20.19	±	4.86	^b^	20.85	±	2.40	^b^	1.83	±	2.02	^a^	4.45	±	2.89		5.02	±	3.25		3.95	±	3.10	
Sugars (g)	1.34	±	4.50		0.30	±	0.43		0.56	±	1.05		1.63	±	5.00		0.07	±	0.12		1.44	±	2.00		0.80	±	0.50	^a^	1.18	±	1.03	^a^	3.59	±	2.62	^b^
Protein (g)	1.98	±	4.09	^a^	0.86	±	1.83	^a^	24.56	±	3.42	^b^	1.59	±	2.84	^a^	1.42	±	2.95	^a^	24.81	±	3.41	^b^	4.67	±	3.64	^a^	5.72	±	3.82	^a^	9.13	±	3.33	^b^
Salt (g)	1.70	±	0.50	^a^	2.02	±	0.26	^b^	1.70	±	0.53	^a^	2.06	±	0.61		2.10	±	0.36		2.04	±	1.05		0.80	±	0.38		1.08	±	0.37		1.06	±	0.67	
Nutri-Score	19.06	±	4.10	^b^	21.07	±	1.96	^c^	15.17	±	3.00	^a^	20.13	±	4.27	^b^	20.38	±	2.90	^b^	14.96	±	3.21	^a^	11.62	±	5.28	^ab^	14.13	±	3.81	^b^	11.19	±	3.36	^a^
**Nutrient Criteria**	**Pb Feta** **2019 (*n* = 8)**				**Pb Feta** **2021 (*n* = 13)**				**Feta** **(*n* = 16)**				**Pb Mozzarella** **2019 (*n* = 12)**				**Pb Mozzarella** **2021 (*n* = 16)**				**Mozzarella** **(*n* = 15)**															
Energy (kcal)	276.4	±	84.3		285.5	±	91.5		243.6	±	58.7		269.2	±	70.4		249.0	±	50.2		264.3	±	43.2													
Fat (g)	21.85	±	7.66		24.65	±	10.04		18.73	±	7.05		21.11	±	5.85		20.50	±	4.13		19.24	±	3.92													
Saturated fat (g)	14.00	±	8.71		16.65	±	8.64		11.74	±	4.30		14.90	±	5.62		14.92	±	6.30		12.13	±	2.54													
Carbohydrate (g)	12.70	±	10.34	^b^	9.95	±	5.74	^b^	1.75	±	1.38	^a^	14.56	±	7.09	^b^	13.74	±	6.58	^b^	2.31	±	2.10	^a^												
Sugars (g)	0.75	±	1.09		0.77	±	1.18		1.65	±	1.43		0.31	±	0.65		0.63	±	1.02		1.03	±	0.92													
Protein (g)	6.84	±	6.96	^a^	5.64	±	5.49	^a^	16.65	±	2.79	^b^	2.87	±	3.14	^a^	1.99	±	2.30	^a^	20.08	±	5.85	^b^												
Salt (g)	1.89	±	0.65	^a^	1.93	±	0.52	^a^	2.50	±	0.47	^b^	1.94	±	1.12	^b^	1.56	±	0.50	^ab^	0.96	±	0.58	^a^												
Nutri-Score	16.88	±	6.20		18.69	±	5.04		15.44	±	3.08		18.42	±	5.73	^b^	17.25	±	6.11	^b^	11.47	±	2.59	^a^												

* Calculated mean values from AUSNUT, Fineli, USDA, and BLS databases; ^a–c^ values within a food group with differing superscript letters are statistically significantly different (*p* ≤ 0.05).

**Table 7:** Coverage of the daily mineral requirements by pb alternative products compared to animal-based products *. Percentage related to the recommendations of the D-A-CH reference values, given in mg per 100 g of foods, and related to gender and age groups (19–25 Y (years) and ≥65 Y).

				Cheese (*n* = 53)		Pb Cheese (*n* = 4)			Meat Burger (*n* = 26)		Pb Burger (*n* = 4)			Meat Salami (*n* = 23)		Pb Salami (*n* = 4)			Meat Sausage (*n* = 37)		Pb Sausage (*n* = 4)		
				Mean (%)					Mean (%)					Mean (%)					Mean (%)				
D-A-CH reference values—female	AG1 ^1^	AG1+ ^2^	AG2 ^3^	AG1	AG2	AG1	AG1+	AG2	AG1	AG2	AG1	AG1+	AG2	AG1	AG2	AG1	AG1+	AG2	AG1	AG2	AG1	AG1+	AG2
Calcium	1000			76.17		31.93			3.18		25.14			1.49		23.35			2.07		20.71		
Copper	1.25			3.02		85.94			7.46		85.43			6.16		75.63			8.96		72.35		
Iron	15	27	10	2.08	3.13	19.53	10.85	29.29	12.98	19.46	29.13	16.18	43.70	5.65	8.48	25.82	14.35	38.73	11.99	17.98	42.28	23.49	63.42
Potassium	4000			2.31		1.41			7.87		11.11			6.05		9.81			8.03		8.13		
Magnesium	310		300	9.21	9.51	14.51		14.99	8.48	8.76	26.48		27.36	5.51	5.70	30.73		31.75	8.29	8.56	29.24		30.21
Sodium	1500			46.08		65.34			26.20		47.80			61.03		92.12			98.57		144.52		
Zinc	8	12		43.70		20.49	13.66		41.95		49.30	32.87		18.61		40.40	26.94		38.59		34.81	23.21	
Phosphorus	700			71.38		15.77			23.76		42.14			22.75		40.02			27.82		38.52		
D-A-CH reference values—male	AG1 ^1^	AG1+ ^2^	AG2 ^3^	AG1	AG2	AG1	AG1+	AG2	AG1	AG2	AG1	AG1+	AG2	AG1	AG2	AG1	AG1+	AG2	AG1	AG2	AG1	AG1+	AG2
Iron	10	18		3.13		29.29	16.27		19.46		43.70	24.28		8.48		38.73	21.52		17.98		63.42	35.23	
Magnesium	400		350	7.13	8.15	11.24		12.85	6.57	7.51	20.52		23.45	4.27	4.88	23.82		27.22	6.42	7.34	22.66		25.90
Zinc	14	21		24.97		11.71	7.80		23.97		28.17	18.78		10.63		23.09	15.39		22.05		19.89	13.26	

* Calculated mean values from AUSNUT, Fineli, USDA, and BLS databases; ^1^ AG1 = age group 19–25 Y; ^2^ AG1+ = age group 19–25 Y, recommendation for vegetarian diets due to lower bioavailability [31] calculated for iron +80% and zinc +50%; ^3^ AG2 = age group ≥ 65 Y.

**Table 8:** Coverage of the daily vitamin requirements by pb alternative products compared to animal-based products *. Percentage related to the recommendations of the D-A-CH reference values, given in mg ^#, ##^ per 100 g of foods, and related to gender and age groups (19–25 Y (years) and ≥65 Y).

			Cheese (*n* = 53)		Pb Cheese (*n* = 4)		Meat Burger (*n* = 26)		Pb Burger (*n* = 4)		Meat Salami (*n* = 23)		Pb Salami (*n* = 4)		Meat Sausage (*n* = 37)		Pb Sausage (*n* = 4)	
			Mean (%)				Mean (%)				Mean (%)				Mean (%)			
D-A-CH reference values—female	AG1 ^1^	AG2 ^2^	AG1	AG2	AG1	AG2	AG1	AG2	AG1	AG2	AG1	AG2	AG1	AG2	AG1	AG2	AG1	AG2
Vitamin B_1_	1		3.00		1.62		16.46		5.22		40.24		11.87		32.96		6.90	
Vitamin B_2_	1.1	1	32.44	35.69	7.66	8.43	15.61	17.17	18.86	20.74	18.94	20.84	21.42	23.56	14.56	16.02	24.70	27.17
Vitamin B_3_	13	11	1.73	2.05	1.35	1.60	40.24	47.56	14.88	17.59	46.10	54.48	12.07	14.27	37.50	44.32	7.96	9.40
Pantothenic acid	6		7.33		1.45		6.25		6.47		6.67		5.22		6.00		3.96	
Vitamin B_6_	1.4		4.57		5.74		17.49		38.37		30.06		34.47		21.31		20.51	
Folate ^#^	300		8.48		5.04		6.10		19.67		0.91		16.74		1.73		14.98	
Biotin ^##^	40		4.90		4.17		10.06		41.47		3.80		57.11		3.85		39.86	
Vitamin B_12_^##^	4		41.01		65.55		42.25		4.38		40.45		4.99		18.96		2.63	
Vitamin C	95		0.16		95.09		0.69		17.91		2.16		nr		3.83		2.21	
Vitamin A ^#^	700		31.79		0.05		2.53		0.11		1.09		0.06		0.94		nr	
Vitamin D ^##^	20		1.81		nr		1.43		nr		2.21		nr		1.46		nr	
Vitamin E	12	11	3.42	3.73	4.92	5.37	3.64	3.73	16.50	5.37	4.02	4.38	49.63	54.14	2.90	3.16	61.91	67.54
Vitamin K ^##^	60	65	9.67	8.93	5.79	5.34	9.15	8.93	24.92	5.34	6.94	6.41	32.88	30.35	7.68	7.09	38.93	35.94
D-A-CH reference values—male	AG1 ^1^	AG2 ^2^	AG1	AG2	AG1	AG2	AG1	AG2	AG1	AG2	AG1	AG2	AG1	AG2	AG1	AG2	AG1	AG2
Vitamin B_1_	1.3	1.1	2.31	2.72	1.24	1.68	12.66	14.97	4.02	2.91	30.95	36.58	9.13	10.80	25.35	29.96	5.31	6.28
Vitamin B_2_	1.4	1.3	25.49	27.45	6.02	9.62	12.26	13.21	14.82	9.45	14.88	16.03	16.83	18.12	11.44	12.32	19.40	20.90
Vitamin B_3_	16	14	1.41	1.61	1.10	2.83	32.70	37.37	12.09	15.56	37.46	42.81	9.81	11.21	30.47	34.82	6.46	7.39
Vitamin B_6_	1.6		4.00		5.03		15.30		33.57		26.30		30.17		18.64		17.95	
Vitamin C	110		0.13		82.12		0.60		15.47		1.87		nr		3.31		1.91	
Vitamin A ^#^	850	800	26.18	27.82	0.04	0.04	2.08	2.21	0.09	0.10	0.89	0.95	0.05	0.05	0.77	0.82	nr	nr
Vitamin E	15	12	2.74	3.42	3.94	4.92	2.91	3.64	13.20	16.50	3.21	4.02	39.70	49.63	2.32	2.90	49.53	61.91
Vitamin K ^##^	70	80	8.29	7.25	4.96	4.34	7.85	6.87	21.36	18.69	5.95	5.21	28.18	24.66	6.58	5.76	33.37	29.20

* Calculated mean values from AUSNUT, Fineli, USDA, and BLS databases; ^#^ μg equivalents, ^##^ μg; ^1^ AG1 = age group 19–25 Y; ^2^ AG2 = age group ≥ 65 Y; nr = not reported.

## Data Availability

Data from measurements are available upon request from the corresponding author.
